# Metronidazole-Induced Encephalopathy in a Patient With Metastatic Cancer

**DOI:** 10.7759/cureus.102331

**Published:** 2026-01-26

**Authors:** Anish K Sethi, Elizabeth O'Connor, Kenil Upadhyay, Kyra Valent, Ruchika Darapaneni, Nisha Busch, Kendal Marshall, Dewmi S Arachchi, Cassiopeia Roychowdhury

**Affiliations:** 1 School of Medicine, Drexel University College of Medicine, West Reading, USA; 2 Family Medicine, Tower Health Reading Hospital, West Reading, USA; 3 Psychiatry, Tower Health Reading Hospital, West Reading, USA

**Keywords:** encephalopathy, encephalopathy in cancer patient, long-term antibiotics, medication adverse effect, metastatic cancer, metronidazole-induced encephalopathy

## Abstract

Metronidazole-induced encephalopathy (MIE) is a rare adverse effect from long-term metronidazole use. We describe a patient with metastatic cancer and complicated abscesses on long-term antibiotics who presented to the emergency department with acute encephalopathic features including reported weakness and confusion. Physical examination was remarkable for chronic right-sided weakness from a previous stroke and reported abnormal right-sided finger-to-nose testing, which may have been due to weakness from his prior stroke. Head CT was non-contributory, and brain MRI showed bilateral edema in cerebellar dentate nuclei, suggestive of MIE. We recommend having a low threshold for brain MRI in patients on long-term metronidazole to rule out other diagnoses and, in patients with lasting neurologic deficits, early neurology and palliative care involvement to improve quality of life.

## Introduction

Encephalopathy describes neural dysfunction from a variety of underlying etiologies. Primary neurologic causes of encephalopathy refers to neurogenic dysfunction from infection, inflammation, or vascular causes, whereas systemic encephalopathy encompasses metabolic derangements or organ dysfunction culminating in an encephalopathic state. One subtype of systemic encephalopathy is drug-induced encephalopathy, which may present in an acute or subacute manner [1]. Although infrequent, antibiotics, such as metronidazole, can cause systemic encephalopathy [2,3]. Most cases have been reported in the USA with a higher proportion of patients being male and over eighteen years of age [3]. Long-term use of metronidazole can cause neurologic deficits, and patients can present with neurologic symptoms at a broad range of cumulative doses, possibly due in part to impaired excretion from hepatic failure [3-7]. The pathophysiology of metronidazole-induced encephalopathy (MIE) is not currently known [8]. Patients often experience cerebellar, brainstem, encephalopathic, and seizure symptoms, and, in most cases, metronidazole-induced encephalopathy (MIE) is confirmed by MRI showing hyper-intensities in the cerebellar dentate nuclei [2,3,9]. Despite discontinuation of the drug upon recognition of neurologic symptoms, outcomes are mixed, as some patients may experience lifelong neurologic deficits or death, whereas others may fully recover [3,10].

We describe a 62-year-old male with multiple severe comorbidities who presented with encephalopathic features that led to classic MRI findings indicative of MIE. Subsequent discontinuation and adjustment of his antibiotic regimen for intra-abdominal abscesses led to improvement in symptoms.

## Case presentation

A 62-year-old male with metastatic colon adenocarcinoma on palliative chemotherapy presented to the emergency department (ED) one and a half months prior to admission for MIE. During this initial hospitalization, he met systemic inflammatory response syndrome criteria, and CT imaging revealed bilateral intra-abdominal abscesses. He was started on piperacillin-tazobactam, with adjustments made based on cultures. Cultures from the left lower quadrant collection revealed resistant *Streptococcus mitis, Bacteroides fragilis*, and other organisms. His antibiotic regimen was modified to intravenous vancomycin, ceftriaxone, and oral metronidazole. Oral metronidazole 500 milligrams was to be taken every eight hours. Despite the potential interaction between metronidazole and 5-florouracil, a component of his chemotherapy regimen, infectious disease specialists determined that metronidazole was essential for optimal treatment. His last chemotherapy was the month prior to this admission, and chemotherapy was held while he was receiving antibiotics for his abdominal infections. His antibiotic regimen was adjusted on discharge, and he was discharged with an abdominal drain in place.

A couple weeks after abdominal drain placement, the patient returned to the ED with chest pain and increased left-sided pleural effusion and atelectasis. Intravenous Vancomycin and oral metronidazole were continued and a switch was made to ceftriaxone from an outpatient oral antibiotic for better pneumonia coverage at that time. During this hospitalization, cultures from a left upper quadrant (LUQ) collection revealed *Candida krusei, Pseudomonas aeruginosa, Saccharomyces cerevisiae,* gram-positive organisms, and occasional yeast, without anaerobes. Micafungin was added to his regimen of vancomycin, ceftriaxone, and metronidazole, and several antibiotic adjustments were made as needed. On discharge, approximately one month before his encephalopathic event, the patient was instructed to continue daily intravenous daptomycin and micafungin, start oral levofloxacin, and continue metronidazole. By this time, metronidazole had been in use for nearly one month since his initial hospitalization. 

The patient presented again to the ED weeks later, now with increasing weakness, confusion, and subjective color change in his percutaneous drainage bag. His caregiver described one to two weeks of lethargy and difficulty with ambulation followed by two days of confusion and slurred speech, which was different from his baseline. He also had a fall the week before admission. On physical exam, the patient had chronic right-sided weakness from a stroke 3 years prior and reported abnormal right-sided finger-to-nose testing, although the latter may also have been due to weakness from his prior stroke. A chest X-ray revealed an improved left lung base opacity, non-contrast head CT showed chronic gliosis and encephalomalacia from a prior stroke, with no acute changes, and cervical spine CT was non-contributory. A CT scan of the chest, abdomen, and pelvis showed a small pleural effusion, minimal changes in LUQ fluid collection, and progression of peritoneal cancer. 

**Figure 1:**
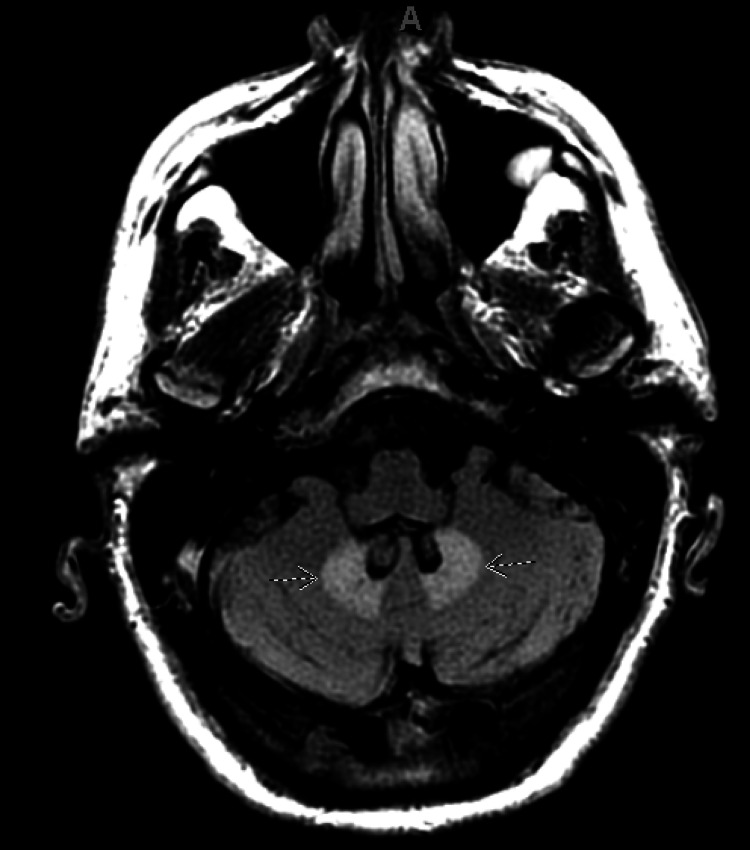
T2 FLAIR Brain MRI Without Contrast. Brain MRI of the patient revealed T2 FLAIR bilateral hyperintensity in the dentate nuclei of the cerebellum (arrows).

**Figure 2:**
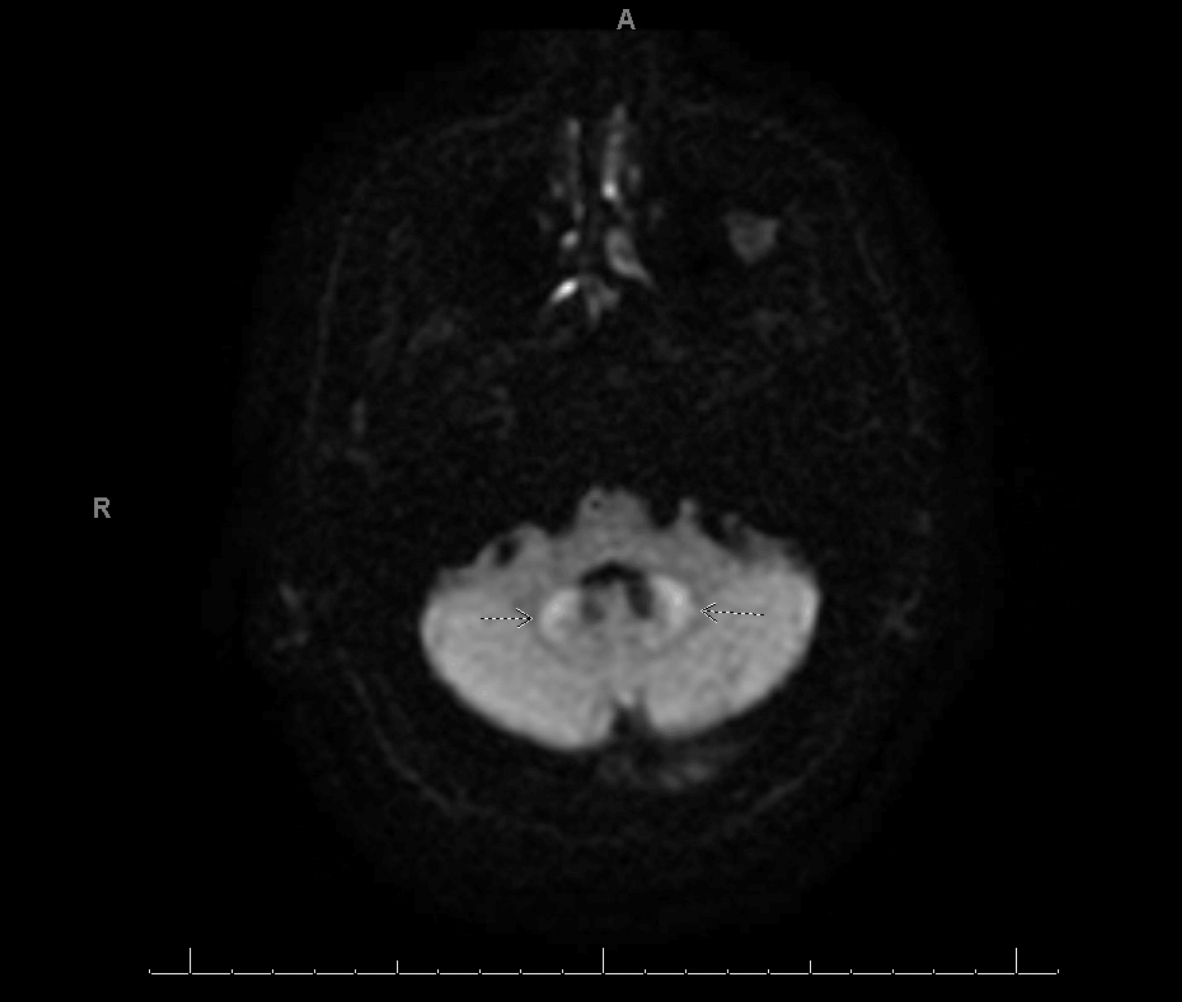
DWI Sequence Brain MRI. Brain MRI of this patient revealed T2 FLAIR bilateral hyperintensity in the dentate nuclei of the cerebellum (Figure 1) along with a bright signal on DWI (arrows on Figure 2).

**Figure 3:**
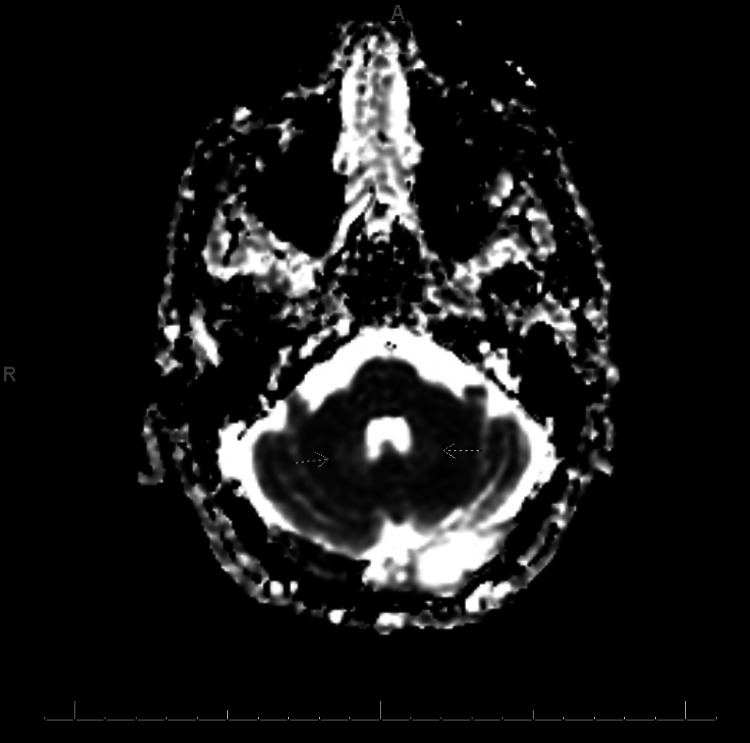
ADC Sequence Brain MRI. Brain MRI of this patient revealed T2 FLAIR bilateral hyperintensity in the dentate nuclei of the cerebellum (Figure 1) along with a bright signal on ADC (arrows on Figure 3).

Given the patient's history, multiple differential diagnoses were considered, including brain metastasis, multifactorial encephalopathy from elevated lactic acid, and, less likely, stroke or transient ischemic attack. Due to concern for brain metastasis, a brain MRI was ordered. MRI revealed a chronic infarct (from a prior known stroke) with diffuse cortical petechial hemorrhage and bilateral T2 FLAIR signal hyperintensity without atrophy in the dentate nuclei (Figures 1-3), typical of non-specific edema and consistent with long-term metronidazole use. There were no noted brain metastases on MRI. At that time, metronidazole was discontinued, and the patient was transitioned to an antibiotic regimen including amoxicillin-clavulanate, posaconazole, and levofloxacin. Over the next few days, his neurologic symptoms, including confusion, gradually improved. He was discharged home on this regimen for his persistent abdominal abscesses. At his post-hospital visit one week after discharge, the patient reported improved appetite, resolved confusion, and gradually improving mobility. These imaging findings are consistent with T2 shine through, and along with the patient's symptoms and presentation, this presentation is highly suggestive of metronidazole-induced encephalopathy. 

## Discussion

The classic picture of MIE involves long-term metronidazole therapy with subsequent encephalopathy without an obvious cause until an MRI is ordered [3]. One review article calculated a median of 35 days on metronidazole in MIE patients, and 90% of them had hyperintensities in cerebellar dentate nuclei on T2 FLAIR MRI [3]. Our patient was prescribed 500 mg oral metronidazole every 8 hours for approximately 50 days and developed confusion and weakness suggestive of encephalopathy over a period of days to weeks. Brain MRI revealed bilateral edema in the dentate nuclei on MRI, which was consistent with the typical patient presentation for MIE. Due to the multiple factors, comorbidities, and polypharmacy in our patient, we cannot definitively conclude that our patient’s encephalopathy was due to metronidazole despite most evidence supporting MIE. However, this patient’s acute encephalopathic symptoms improved after discontinuation of metronidazole, supporting MIE as the etiology of his symptoms.

Despite many case reports and reviews on MIE, very few studies have described this phenomenon in a patient with cancer. Many diagnoses are possible in a patient with cancer presenting with confusion, including typical causes of encephalopathy and diagnoses that are directly related to cancer and its treatment [11]. In an encephalopathic patient with cancer taking metronidazole for any amount of time, we suggest having a low threshold for ordering an MRI, as some patients develop MIE as soon as one week after metronidazole initiation, whereas others develop it after months [3]. MRI can detect vascular, infectious, and metastatic causes [11] and, in patients with an unremarkable CT, can eliminate other causes of encephalopathy (i.e., metastasis). Additionally, a follow-up MRI may not be required if symptoms improve or resolve in the absence of other findings that would otherwise warrant follow-up. For most patients, prompt discontinuation of metronidazole results in resolution of symptoms; however, some patients may have poorer outcomes, and it is not known whether time to discontinuation impacts prognosis. Currently, no factors have been identified that can definitively predict prognosis after stopping metronidazole [3]. Once MIE develops, an antibiotic regimen without metronidazole must be utilized.

Encephalopathy in a patient with cancer presents a new array of considerations. In our patient, many of his acute deficits improved. However, in patients with residual neurologic deficits, new-onset symptoms may decrease patients’ quality of life. In those cases, it is imperative to involve neurology and palliative care to optimize care and reduce symptomatic burden on the patient. Future studies can determine factors associated with improved or worsened prognosis in addition to best practices for patient care in patients with cancer. 

## Conclusions

Given the unknown prognosis of MIE, acute encephalopathy in patients with cancer on metronidazole for long-term abscesses should prompt providers to obtain an MRI shortly after patient presentation to rule out distant metastasis and evidence of additional pathologies, including MIE. Persistent symptoms after cessation of metronidazole in patients with MIE can assist providers in assessing other possible etiologies of encephalopathy. Palliative care is an underrecognized and underutilized resource that should be involved in cases of patients with cancer diagnosed with MIE to assist with lasting neurologic deficits.
